# Outcomes assessment in intrahepatic cholangiocarcinoma using qualitative and quantitative imaging features

**DOI:** 10.1186/s40644-020-00323-0

**Published:** 2020-07-03

**Authors:** Michael J. King, Stefanie Hectors, Karen M. Lee, Olamide Omidele, James S. Babb, Myron Schwartz, Parissa Tabrizian, Bachir Taouli, Sara Lewis

**Affiliations:** 1 Department of Diagnostic, Molecular and Interventional Radiology, Icahn School of Medicine at Mount Sinai, One Gustave L. Levy Place, Box 1234, New York, NY 10029-6574 USA; 2 BioMedical Engineering and Imaging Institute, Icahn School of Medicine at Mount Sinai, New York, NY USA; 3 Department of Radiology, Weill Cornell Medicine, New York, NY USA; 4 Department of Radiology, New York University Langone Medical Center, New York, NY USA; 5 Recanati/Miller Transplantation Institute, Icahn School of Medicine at Mount Sinai, New York, NY USA

**Keywords:** Bile duct cancer, Texture, Magnetic resonance imaging (MRI), Computed tomography (CT), Survival

## Abstract

**Background:**

To assess the performance of imaging features, including radiomics texture features, in predicting histopathologic tumor grade, AJCC stage, and outcomes [time to recurrence (TTR) and overall survival (OS)] in patients with intrahepatic cholangiocarcinoma (ICC).

**Methods:**

Seventy-three patients (26 M/47F, mean age 63y) with pre-operative imaging (CT, *n* = 37; MRI, *n* = 21; CT and MRI, *n* = 15] within 6 months of resection were included in this retrospective study. Qualitative imaging traits were assessed by 2 observers. A 3rd observer measured tumor apparent diffusion coefficient (ADC), enhancement ratios (ERs), and Haralick texture features. Blood biomarkers and imaging features were compared with histopathology (tumor grade and AJCC stage) and outcomes (TTR and OS) using log-rank, generalized Wilcoxon, Cox proportional hazards regression, and Fisher exact tests.

**Results:**

Median TTR and OS were 53.9 and 79.7 months. ICC recurred in 64.4% (47/73) of patients and 46.6% (34/73) of patients died. There was fair accuracy for some qualitative imaging features in the prediction of worse tumor grade (maximal AUC of 0.68 for biliary obstruction on MRI, *p* = 0.032, observer 1) and higher AJCC stage (maximal AUC of 0.73 for biliary obstruction on CT, *p* = 0.002, observer 2; and AUC of 0.73 for vascular involvement on MRI, *p* = 0.01, observer 2). Cox proportional hazards regression analysis showed that CA 19–9 [hazard ratio (HR) 2.44/95% confidence interval (CI) 1.31–4.57/*p* = 0.005)] and tumor size on imaging (HR 1.13/95% CI 1.04–1.22/*p* = 0.003) were significant predictors of TTR, while CA 19–9 (HR 4.08/95% CI 1.75–9.56, *p* = 0.001) and presence of metastatic lymph nodes at histopathology (HR 2.86/95% CI 1.35–6.07/*p* = 0.006) were significant predictors of OS. On multivariable analysis, satellite lesions on CT (HR 2.79/95%CI 1.01–7.15/*p* = 0.032, observer 2), vascular involvement on MRI (HR 0.10/95% CI 0.01–0.85/*p* = 0.032, observer 1), and texture feature MRI variance (HR 0.55/95% CI 0.31–0.97, *p* = 0.040) predicted TTR once adjusted for the independent predictors CA 19–9 and tumor size on imaging. Several qualitative and quantitative features demonstrated associations with TTR, OS, and AJCC stage at univariable analysis (range: HR 0.35–19; *p* < 0.001–0.045), however none were predictive of OS at multivariable analysis when adjusted for CA 19–9 and metastatic lymph nodes (*p* > 0.088).

**Conclusions:**

There was reasonable accuracy in predicting tumor grade and higher AJCC stage in ICC utilizing certain qualitative and quantitative imaging traits. Serum CA 19–9, tumor size, presence of metastatic lymph nodes, and qualitative imaging traits of satellite lesions and vascular involvement are predictors of patient outcomes, along with a promising predictive ability of certain quantitative texture features.

## Background

Mass-forming intrahepatic cholangiocarcinoma (ICC), the most common subtype of ICC (followed by periductal infiltrating and intraductal growth subtypes), is an epithelial malignancy of the intrahepatic bile ducts that is typically associated with poor patient outcomes; as less than 40% of patients with resectable ICC survive more than 5 years, and those with unresectable disease typically survive less than 12 months [1–4]. Although the incidence of ICC is highest in Asia, a rise in known risk factors (such as chronic viral hepatitis, cirrhosis, primary sclerosing cholangitis, fibropolycystic liver disease, and recurrent pyogenic cholangitis) has led to a worldwide rise in its incidence and mortality over the past two decades [1, 5–8]. In the United States (US), the reported average incidence of ICC has increased from 0.44 to 1.18 cases per 100,000, representing an annual percentage change of 2.3% between 1973 and 2012 [9].

Despite liver resection followed by adjuvant chemoradiation therapy being the most effective treatment, documented postoperative recurrence rates reach as high as 53 to 79%, and most patients die of their disease [10–12]. These dismal facts highlight the need for improved noninvasive tumor characterization and enhanced risk stratification in an effort to better predict clinical outcomes and augment perioperative management, including initiating adjuvant chemotherapy. Histopathologic findings of tumor size, tumor grade, intrahepatic metastasis, vascular invasion, and lymph node metastasis have been established as poor independent prognostic factors in ICC [2, 13].

Recent studies have investigated the role of cross-sectional imaging for the characterization of ICC pathology and outcomes. The degree of enhancement on delayed phase computed tomography (CT) was shown to correlate with the amount of fibrous stroma and the frequency of perineural invasion, both of which are poor independent prognostic indicators [14]. Conversely, arterial enhancement of ICC on CT has been shown to be an independent predictor of improved survival [15]. In a study with emphasis on diffusion-weighted imaging (DWI) measured with magnetic resonance imaging (MRI), the authors demonstrated that ICCs with > 1/3 diffusion restriction had more favorable histopathologic features and better clinical outcomes compared to those with < 1/3 diffusion restriction, as less diffusion restriction is believed to correlate with more fibrous stroma [16]. With regards to the apparent diffusion coefficient (ADC) quantification, it has been suggested that the ADCmean of ICC is significantly lower than that of the adjacent liver parenchyma, and that poorly differentiated tumors demonstrated a significantly lower ADCmean than well or moderately differentiated tumors [17, 18]. Lastly, it is recognized that F-18 FDG PET/CT is an important diagnostic tool in staging of ICC. Recent studies have described SUV (standardized uptake value) quantification as a significant discriminant parameter for predicting poorer outcomes [19, 20].

Radiomics is a process by which one can extract quantitative data containing valuable information about pathophysiology from digital medical images [21]. There is very limited data assessing the role of radiomics in ICC. To the best of our knowledge, only one published study has shown an association between texture features based on CT and expression of tumor markers of hypoxia in ICC [22].

While all these reports acknowledging the imaging characterization of ICC are promising, work assessing the relationship between imaging parameters and clinical outcomes is lacking. The main objectives of our study were to assess the diagnostic performance of imaging features, including quantitative radiomics texture features, in determining histopathologic tumor grade, AJCC stage, and in predicting outcomes [time to recurrence (TTR) and overall survival (OS)] in comparison with multiple clinicopathologic and demographic variables in patients with ICC.

## Methods

### Patients

**Fig. 1:**
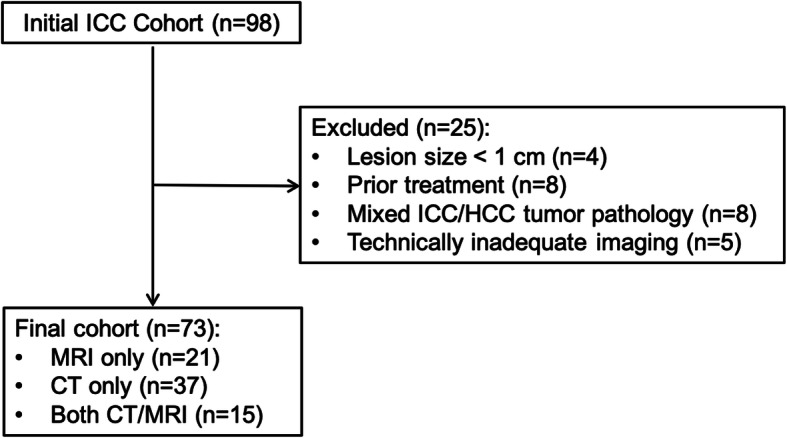
Study flowchart of patient population

This retrospective single-center study was approved by the local institutional review board at the Icahn School of Medicine at Mount Sinai (ISMMS), New York, NY, with exemption for patient consent. The ISMMS Department of Surgery electronic database was queried between August 2003 and January 2017 using the search term “cholangiocarcinoma” and “CT” and/or “MRI”. Inclusion criteria were: 1) patients with pathologically proven mass-forming ICC, 2) patients who underwent preoperative multiphasic CT, MRI, or both within 6 months prior to resection (segmentectomy or partial hepatectomy), 3) lesion size ≥1 cm, and 4) no interval therapy between imaging and surgery. Exclusion criteria were: 1) lesion size < 1 cm, 2) patients who had undergone prior locoregional or systemic treatment for their malignancy, 3) mixed ICC/hepatocellular carcinoma (HCC) histology, and 4) patients with technically inadequate imaging studies. Of the initially included patients (*n* = 98), twenty-five patients were excluded as follows: lesion size < 1 cm (*n* = 4), prior treatment (*n* = 8), mixed ICC/HCC tumor pathology (*n* = 8), and technically inadequate imaging (*n* = 5). The final study population comprised 73 patients (26 M/47F; mean age 63 ± 11.4 years; range 24–81 years). The study flow chart is shown in Fig. 1.

The following clinical data was recorded for each patient at the time of preoperative imaging after interrogating the medical records: age, gender, race/ethnicity, serum CA 19–9, and presence and etiology of underlying chronic liver disease, presence/absence of tumor recurrence, tumor recurrence date, and date of death or date of last follow up.

### Image acquisition

Multiphasic CT and/or MRI were performed using a variety of clinically available imaging platforms, as outside institutional imaging studies comprised some of our preoperative study population. On CT, these included GE Medical Systems, Siemens and Philips scanners. Several multichannel MRI systems were used for scanning, including 1.5 T (Avanto, Aera, Sonata, and Symphony, Siemens Healthineers; and Signa HD, HDxt, Optima 450w, GE Medical Systems) (*n* = 32) or 3 T (Skyra, Siemens Healthineers, 750, GE Medical Systems) (*n* = 4) imaging platforms.

The sequences and acquisition parameters varied slightly between different imaging platforms, however arterial phase (AP) images were obtained 20–40 s after iodinated (CT) or gadolinium-based (MRI) intravenous contrast administration and portal venous phase (PVP) images were obtained 60–100 s after contrast administration. Twelve MRI exams were performed with a liver specific gadolinium based contrast agent (gadoxetic acid, Eovist/Primovist, Bayer Healthcare); in these cases, equilibrium (EP)/transitional phase (TP) images were obtained after 3–6 min of contrast administration, and hepatobiliary phase (HBP) images were obtained 10 to 20 min after contrast administration. Extracellular contrast agents used in the remaining cases included gadobutrol (Gadavist, Bayer Healthcare) and gadopentetate dimeglumine (Magnevist, Bracco Diagnostics). Diffusion weighted imaging (DWI) was available in 19 patients, with b-values ranging from 50 to 1000 s/mm^2^. ADC maps were generated automatically by the scanner.

### Qualitative image analysis

For qualitative analysis, two fellowship-trained, board-certified abdominal radiologists (observer 1, SL; and observer 2, KL, with 8 and 13 years of experience in abdominal imaging at the time of the study, respectively) independently reviewed the CT and MR images using PACS (Centricity 3.0, General Electric Medical Systems). The reviewers were aware that the patients had ICC, however were unaware of any other clinicopathologic information. The index lesion, identified as the largest lesion on a single axial image and selected by both observers in consensus, was used for qualitative and quantitative analysis and for correlation with pathology findings and outcomes. In patients with multifocal ICC (*n* = 4), the single largest lesion was analyzed; multifocal ICC was defined as the presence of at least one additional tumor nodule greater than 2 cm away from the index lesion.

**Fig. 2:**
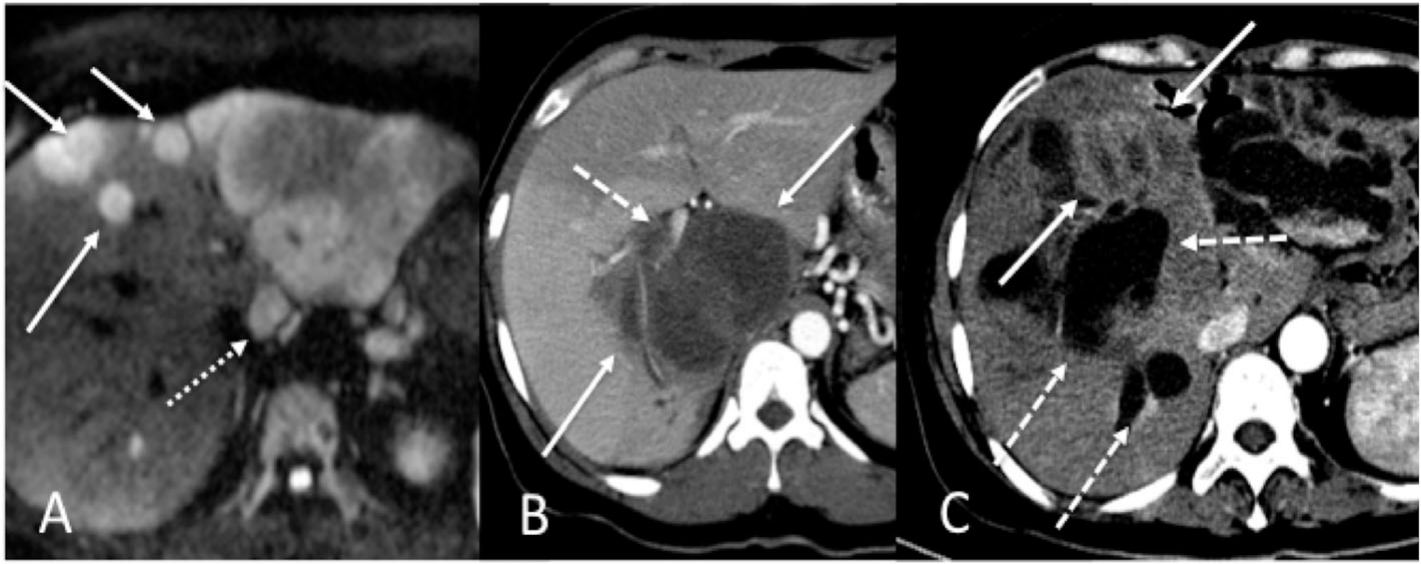
Illustrative examples of qualitative imaging traits of ICC. **a** 46-year-old female with moderately differentiated ICC (AJCC stage 3). Axial high b-value (b = 800) diffusion-weighted image demonstrates a dominant tumor in left lateral hepatic lobe with multiple satellite nodules (defined as smaller tumor lesions within 2 cm of the dominant mass) (arrows) and metastatic lymphadenopathy (dashed arrow) adjacent to the liver. **b** 37-year-old female with moderately differentiated ICC (AJCC stage 1). CT acquired during the arterial phase demonstrates a large hypovascular caudate lobe and right hepatic lobe tumor (arrows) and associated right portal vein invasion (dashed arrow). **c** 48-year-old male with poorly differentiated ICC (AJCC stage 3). CT acquired during the arterial phase demonstrates a left hepatic lobe tumor (solid arrows) causing marked biliary obstruction (dashed arrows)

The observers recorded the segmental location of the index lesion on PVP, as well as the presence/absence of ancillary findings including liver capsule bulging or retraction (unequivocal outward or inward liver contour change immediately superficial to an ICC lesion, respectively), vascular involvement (the presence of obvious enhancing tumor thrombus within the portal and/or hepatic veins, vascular encasement or distortion), peripheral biliary ductal dilatation, satellite lesions (small tumor nodules within 2 cm of the index lesion), and presence of additional non-satellite lesions (Fig. 2). Presence or absence of liver cirrhosis based on established morphologic criteria was recorded [23].

Dynamic enhancement patterns on CT and MRI were classified into 2 categories to allow for adequate statistical analysis: peripheral progressive whole-lesion (progressive whole-lesion enhancement starting from its periphery over time) + persistent rim enhancement, or other (includes wash in/wash out, solid whole lesion progressive (progressive whole-lesion enhancement over time), hypovascular, and necrotic). ICCs were categorized on T2-weighted imaging as hyperintense to adjacent liver parenchyma, targetoid (T2 hyperintense peripheral cellular region with a more T2 hypointense central core), or other (includes isointense and heterogeneous). ICCs were categorized on DWI sequences (when available) as hyperintense to adjacent liver parenchyma, targetoid (DWI hyperintense peripheral cellular region with a more DWI hypointense central core), or other (includes isointense and heterogeneous). Lesions were evaluated on ADC maps as hypointense or other (includes isointense, hyperintense, inverse targetoid, and heterogeneous). For cases performed with gadoxetic acid, lesions were assessed on the T1-weighted HBP as: hypointense compared to surrounding liver parenchyma or other (includes isointense, hyperintense or targetoid).

### Quantitative image analysis

The observers recorded the maximum lesion size of the index lesion on PVP. Additional quantitative image analysis was performed utilizing regions of interest (ROIs) drawn on index lesions on all applicable phases of enhancement and the pre-contrast phase, as well as on non-tumoral liver parenchyma on the PVP by Observer 3 (MK, a fourth-year radiology resident with 1 year of experience in abdominal MRI at the time of the study). A single ROI was drawn to include as much of the index lesion as possible on the axial slice designated by the observers on each phase. In all cases, the ROI diameter was > 1 cm.

ROIs were drawn on the Osirix DICOM viewer (v5.5.2, Pixmeo, Bernex, Switzerland). ROI data were subsequently analyzed with custom written scripts using MATLAB (vR2016b, Mathworks Inc., Natick, MA). Lesion enhancement ratios (ERs) were calculated for arterial and portal venous phases for CT exams, and Ers were calculated for MRI exams for AP, PVP and equilibrium/transitional phases, as well as HBP (when available) as follows:

$$ \mathrm{ER}=\left[\mathrm{mean}\ \mathrm{SI}\ {\left(\mathrm{signal}\ \mathrm{intensity}\right)}_{\mathrm{contrast}\ \mathrm{phase}}-\mathrm{mean}\ {\mathrm{SI}}_{\mathrm{noncontrast}\ \mathrm{phase}}\right]/\left[\mathrm{mean}\ {\mathrm{SI}}_{\mathrm{noncontrast}\ \mathrm{phase}}\right]. $$

Lesion ADCmean and ADCmin values were calculated in 19 patients using monoexponential fitting of the signal intensity (SI) decay curve with the following formula using two b-values: ADC = ln (S2/S1)/(b1-b2), where S1 and S2 are the SI at b-values b1 = 50 s/mm^2^ and b2 = 400–500 s/mm^2^, respectively; these b-values were selected because they were the most common combinations among the different DWI protocols.

Multiple second order Haralick texture features—energy, contrast, correlation, variance, homogeneity, sum average, sum variance, sum entropy, entropy, difference variance, difference entropy, information measure of correlation 1, information measure of correlation 2, and maximal correlation coefficient—were extracted from signal values in the ROIs on PVP images also utilizing MATLAB software for both CT and MRI by observer 4 (SH, an MR physicist with 6 years of experience at the time of the study) in consensus with observer 3 [21, 24–28]. Before texture analysis, SI values in the ROIs were normalized to a range within three standard deviations of the mean SI of the ROI and decimated to 64 discrete bin values. The PVP was selected for texture analysis to allow for adequate lesion conspicuity and for consistency, as PVP images were performed in all MRI and CT cases. Because data from different imaging vendors, platforms, and protocols was included, data pre-processing using normalization was performed to reduce the signal variation between acquisitions [29].

### Study endpoints

#### Histopathologic analysis

Pathologic tumor grade (defined as G1: well differentiated; G2: moderately differentiated; G3: poorly differentiated) and AJCC tumor stage (8th edition) were extracted from pathology reports from the electronic medical record [30–32]. When a single tumor contained regions of different degrees of differentiation, the lesion was classified based on the worse degree of tumor differentiation. Presence/absence of vascular invasion and presence/absence of nodal metastasis within regional lymph nodes submitted with all surgical specimens were also recorded.

#### Patient outcomes

Our study endpoint of time to recurrence (TTR) was defined as the time between surgical resection and the development of locoregional or distant tumor recurrence. Overall survival (OS) was calculated as the time between surgical resection and the date of death (from any cause) or the date of last clinical or imaging follow-up. A final review of the patient’s medical records was undertaken in September 2018.

### Statistical analysis

Data acquired from each imaging modality (CT or MRI) was analyzed separately. For the purpose of statistical analysis, patients with multifocal ICC (*n* = 4) were included in the group designated as positive for satellite lesions.

Logistic regression was used to assess the utility of demographic, clinical, and imaging factors, alone and in combination, as predictors of tumor grade and AJCC stage, and was quantified in terms of area under the ROC curve. In order to perform logistic regression analysis, AJCC stage was analyzed as binary variable as stage I-II vs. III.

The association of each clinical, demographic, qualitative and quantitative imaging factor with OS and TTR was assessed using log-rank and generalized Wilcoxon tests. Survival curves and the median and inter-quartile range of OS and TTR were derived using the Kaplan-Meier product-limit estimator. Cox proportional hazards regression was used to estimate the hazard ratio (HR) of individual factors as predictors of each survival outcome and to assess the effects of feature combinations for the prediction of each outcome. Only variables observed to be significant predictors of at least one outcome according to at least one of the univariable log-rank and Wilcoxon tests were entered in the multivariable analyses. The Fisher exact test was used to assess the association of each qualitative imaging trait from each observer with each binary outcome.

Stepwise variable selection in the context of logistic and Cox proportional hazards regression was then used to identify subsets of variables representing significant independent predictors of each binary and survival outcome, respectively. Inter-observer agreement in terms of the qualitative imaging traits was assessed using the simple kappa (K) coefficient. All statistical tests were conducted at the two-sided 5% significance level using SAS 9.4 (SAS Institute, Cary, NC).

## Results

### Demographic, clinical, histopathologic and outcomes findings

Seventy-three index ICC lesions were assessed in 73 patients. Twenty-one patients had only MRI, 37 patients had only CT, and 15 patients had both MRI and CT. The mean time interval between initial imaging and resection was 32.4 ± 28.1 days [range 4–169.8 days], and the mean duration of clinical follow up (from initial imaging to date of death or latest available postoperative clinical or imaging data) was 1517.3 ± 984.9 days [range 95–4191 days]. Clinicopathologic characteristics are summarized in Table 1. Forty-seven percent (34/73) of patients initially presented with symptoms of jaundice and/or abdominal pain leading to their diagnostic workup; the remaining lesions were discovered incidentally on imaging for unrelated reasons. None of the patients had diffusely infiltrating disease. Twenty-eight percent (21/73) of patients had underlying liver disease (chronic hepatitis B [*n* = 11], chronic hepatitis C [*n* = 7], and recurrent pyogenic cholangitis [*n* = 3]). Four percent (3/73) of patients were cirrhotic. Serum CA 19–9 level was elevated (defined as ≥35 U/mL) in 47% (34/74) of patients. There was ICC recurrence in 64% (47/73) of patients: 17 had intrahepatic tumor recurrence, 8 had extrahepatic tumor recurrence, and 22 had both intra and extrahepatic recurrence. Forty-seven percent (34/73) of patients died. In our cohort, the Kaplan-Meier method estimated the median TTR to be 53.9 months (IQR 73.2 months, range 1.6–99 months) and the median OS to be 79.7 months (IQR 75.4 months, range 1.8–137.3 months).

**Table 1:** Characteristics of the study population (*n* = 73)

Parameter	
Mean age (y)	63 ± 11.4 [24–81]
Gender (M/F)	26 (36%) / 47 (64%)
Race/Ethnicity	
Caucasian	39 (53%)
Asian	21 (29%)
Hispanic	7 (10%)
African American	5 (7%)
Unknown	1 (1%)
Underlying liver disease (*n* = 21)	
HBV	11 (52%)
HCV	7 (33%)
Recurrent pyogenic cholangitis	3 (15%)
Cirrhosis	3
Serum CA 19–9 (U/mL)	4799.8 ± 23,672.4 [1–177,450]
Mean lesion size on imaging (cm)	6.8 ± 3.4 [1.4–16.0]
Pathologic tumor grade	
Well differentiated (G1)	0 (0%)
Moderately differentiated (G2)	34 (47%)
Poorly differentiated (G3)	36 (49%)
Unavailable	3 (4%)
Lymph node metastasis	18 (25%)
AJCC (8th edition) Tumor Stage	
Stage 1	17 (23%)
Stage 2	24 (33%)
Stage 3	32 (44%)

Unless otherwise indicated, data are numbers of patients and data in parentheses are percentages (percent relative to total number in the respective subgroup). Numbers in brackets represent the lower and upper limits of range

Of the 73 ICC lesions, 47% (34/73) were classified as G2 and 49% (36/73) as G3. No well-differentiated (G1) tumors were found in our study, and tumor grade was not available for 3 patients. AJCC (8th edition) tumor stage breakdown was as follows: stage I, 23% (17/73); stage II, 33% (24/73); and stage III, 44% (32/73). Vascular invasion was present in 67% (49/73), including microvascular invasion in 53% (39/73) and macrovascular invasion in 14% (10/73) of cases. Metastatic lymph nodes were discovered at the time of surgical resection in 25% (18/73) of patients.

Elevated serum CA 19–9 level and pathologic findings of vascular invasion, metastatic lymph nodes, and AJCC tumor stage were all predictors of both TTR and OS (all *p*-values < 0.046). These findings are summarized in Table 2. Multivariable Cox regression analysis found that for TTR, CA 19–9 (HR 2.44 [1.31–4.57], *p* = 0.005) remained a significant independent predictor after adjusting for the other factors. For OS, CA 19–9 (HR 4.08 [1.75–9.56], *p* = 0.001) and the presence of metastatic lymph nodes (HR 2.86 [1.35–6.70], *p* = 0.006) remained significant independent predictors after adjusting for the other factors. The remaining demographic and clinical factors, including age, race/ethnicity, and history of underlying liver disease, were not significant predictors for TTR or OS (*p*-values > 0.05) at univariable or multivariable analysis.

**Table 2:** Association of demographic, laboratory and pathologic features with time to recurrence (TTR) and overall survival (OS) and the hazard ratios (HR) from Cox regression to characterize the effect of each feature on outcomes in 73 patients with ICC

		TTR			OS		
		HR	p (Log-Rank)	p (Wilcoxon)	HR	p (Log-Rank)	p (Wilcoxon)
**Demographic & Laboratory**	Age	0.98	0.163	0.482	0.98	0.107	0.221
	Ethnicity	0.61	0.154	0.265	0.54	0.174	0.271
	Gender	0.98	0.934	0.768	0.79	0.504	0.176
	Underlying liver disease	0.58	0.096	0.059	0.63	0.245	0.226
	Serum CA19–9	2.34	**0.005**	**0.004**	3.72	**0.001**	**0.001**
**Pathology**	Tumor grade	0.94	0.842	0.568	1.17	0.665	0.643
	Vascular Invasion	1.75	0.077	**0.024**	2.36	**0.030**	**0.030**
	Metastatic lymph nodes	1.93	**0.039**	0.052	2.49	**0.009**	**0.005**
	AJCC stage	1.68	**0.008**	**0.005**	1.59	**0.046**	**0.028**
	Cirrhosis	0.29	0.198	0.117	0.48	0.457	0.382

Footnote: Confidence limits for the HR are not provided for these features since they represent competing risk factors rather than factors of interest

Significant results are bolded

### Qualitative image analysis

The 73 index lesions were identified in consensus for qualitative analysis, and the qualitative imaging traits assessed are summarized in Table 3. The observers provided an individual independent assessment of each lesion characteristic for each modality, resulting in 52 CT and 36 MRI exams being evaluated in total. They demonstrated moderate to perfect agreement for most qualitative traits assessed (average kappa = 0.53, range 0.29–1.0).

**Table 3:** Qualitative imaging traits of ICC tumors in 73 patients with assessment of inter-observer agreement

		Observer 1 n (%)	Observer 2 n (%)	Kappa
**CT**	Biliary obstruction	27 (52%)	23 (44%)	0.69
	Capsular bulge/retraction	31 (60%)	17 (33%)	0.50
	Enhancement pattern			0.68
	Peripheral progressive	31 (60%)	32 (62%)	
	Persistent rim	6 (12%)	7 (13%)	
	Other^a^	15 (28%)	13 (25%)	
	Satellite lesions^b^	14 (27%)	12 (23%)	0.69
	Vascular involvement	17 (33%)	21 (40%)	0.59
**MRI**	Biliary obstruction	14 (39%)	14 (39%)	0.55
	Capsular bulge/retraction	16 (44%)	7 (19%)	0.47
	Enhancement pattern			0.54
	Peripheral progressive	24 (66%)	27 (75%)	
	Persistent rim	6 (17%)	5 (14%)	
	Other^a^	6 (17%)	4 (11%)	
	Satellite lesions^b^	7 (19%)	5 (14%)	0.60
	Vascular involvement	13 (36%)	9 (25%)	0.57
	ADC (*n* = 19)	5 (26%)	9 (47%)	0.93
	Hypointense	14 (74%)	10 (53%)	
	Other^#^			
	DWI (*n* = 19)			0.66
	Hyperintense	10 (53%)	15 (79%)	
	Targetoid	6 (32%)	4 (21%)	
	Other^c^	3 (16%)	0 (0%)	
	HBP (*n* = 12)			1.00
	Hypointense	12 (100%)	12 (100%)	
	Other^d^	0 (0%)	0 (0%)	
	T2 (*n* = 36)			0.29
	Hyperintense	21 (58%)	24 (67%)	
	Targetoid	8 (22%)	3 (8%)	
	Other^c^	7 (19%)	9 (27%)	

^a^ Other enhancement patterns include wash in/wash out, solid whole lesion progressive enhancement, hypovascular or totally necrotic

^b^ For the purpose of statistical analysis, patients with multifocal ICC (*n* = 4) were included in the group designated as positive for satellite lesions

^c^ Other includes isointense and heterogeneous

^d^ Other includes isointense, hyperintense or targetoid

Only biliary obstruction on MRI was associated with poor tumor differentiation (*p* = 0.032; observer 1). For prediction of AJCC stage, biliary obstruction on CT was associated with higher stage disease (stage I-II vs. stage III) for both observers (*p* = 0.006 and *p* = 0.002, respectively), as was vascular involvement on CT (*p* = 0.043 and *p* = 0.009). The presence of satellite lesions on CT (*p* = 0.022; observer 1) and vascular involvement on MRI (*p* = 0.005; observer 2) were significant for one observer. Based on univariable analysis, these features were then entered into a logistic regression model, yielding fair accuracy for prediction of worse tumor grade (maximal AUC of 0.68 for biliary obstruction at MRI, *p* = 0.032, observer 1) and higher AJCC stage (maximal AUC of 0.73 for biliary obstruction on CT, *p* = 0.002, observer 2; and AUC 0.73 of 0.73 for vascular involvement on MRI, *p* = 0.01, observer 2). The results from logistic regression analysis of qualitative imaging features as predictors of pathologic grade and tumor stage are listed in Table 4.

**Table 4:** Results from logistic regression analysis of qualitative imaging features as predictors of pathologic grade and AJCC tumor stage. AUC, odds ratio (OR) and 95% confidence interval for the OR for the effect of each CT and MRI qualitative imaging feature derived by each observer on each binary outcome are shown

			Observer 1				Observer 2			
**Outcome**	**Modality**	**Feature**	**AUC**	**OR**	**95% CI**	**P**	**AUC**	**OR**	**95% CI**	**P**
**Tumor Grade (G2 vs. G3)**	CT	Biliary Obstruction	0.50	0.99	0.32–3.01	0.982	0.59	2.02	0.64–6.33	0.228
		Capsular Retraction	0.63	0.33	0.10–1.09	0.068	0.63	0.31	0.09–1.06	0.061
		Enhancement Pattern	0.57	5.00	0.54–46.34	0.157	0.55	2.39	0.4213.67	0.329
		Satellite Lesions	0.58	2.37	0.62–9.09	0.207	0.54	1.66	0.42–6.61	0.470
		Vascular involvement	0.53	0.79	0.24–2.60	0.697	0.53	0.76	0.25–2.38	0.643
	MRI	DWI Appearance	0.58	0.50	0.07–3.55	0.488	0.53	0.75	0.11–5.24	0.772
		Biliary Obstruction	**0.68**	**5.00**	**1.15–21.80**	**0.032**	0.65	3.94	0.91–17.01	0.066
		Capsular Retraction	0.60	2.25	0.57–8.82	0.245	0.52	0.75	0.14–3.98	0.736
		Enhancement Pattern	0.53	1.54	0.29–8.18	0.613	0.50	1.07	0.18–6.22	0.939
		Hepatobiliary Phase	0.55	0.64	0.15–2.72	0.546	0.57	0.55	0.13–2.26	0.406
		Satellite Lesions	0.53	1.54	0.29–8.18	0.613	0.53	1.71	0.25–11.78	0.584
		Vascular involvement	0.51	1.09	0.27–4.41	0.903	0.54	1.46	0.32–6.70	0.628
**AJCC Stage (Stage I-II vs stage III)**	CT	Biliary Obstruction	**0.70**	**5.38**	**1.61–17.97**	**0.006**	**0.73**	**7.18**	**2.10–24.57**	**0.002**
		Capsular Retraction	0.53	0.79	0.26–2.42	0.686	0.56	0.58	0.17–1.91	0.368
		Enhancement Pattern	0.59	7.78	0.84–72.11	0.071	0.57	3.75	0.65–21.46	0.138
		Satellite Lesions	**0.65**	**4.81**	**1.26–18.35**	**0.022**	0.60	3.33	0.86–12.99	0.083
		Vascular involvement	**0.64**	**3.51**	**1.04–11.84**	**0.043**	**0.68**	**4.89**	**1.48–16.13**	**0.009**
	MRI	DWI Appearance	0.56	1.67	0.21–13.22	0.629	0.70	5.33	0.62–45.99	0.128
		Biliary Obstruction	0.66	3.86	0.94–15.86	0.061	0.66	3.86	0.94–15.86	0.061
		Capsular Retraction	0.56	0.60	0.16–2.29	0.455	0.56	0.43	0.07–2.58	0.355
		Enhancement Pattern	0.55	1.89	0.36–10.03	0.455	0.58	3.00	0.47–19.04	0.244
		Hepatobiliary Phase	0.66	5.32	0.94–29.99	0.058	0.63	3.55	0.76–16.43	0.106
		Satellite Lesions	0.61	4.09	0.67–24.83	0.126	0.60	6.33	0.63–63.64	0.117
		Vascular involvement	0.63	3.00	0.73–12.27	0.126	**0.73**	**19.00**	**2.03–177.93**	**0.010**

Footnote: Pathologic tumor grade was defined as follows: G2: moderately differentiated, G3: poorly differentiated

Significant results are bolded

**Fig. 3:**
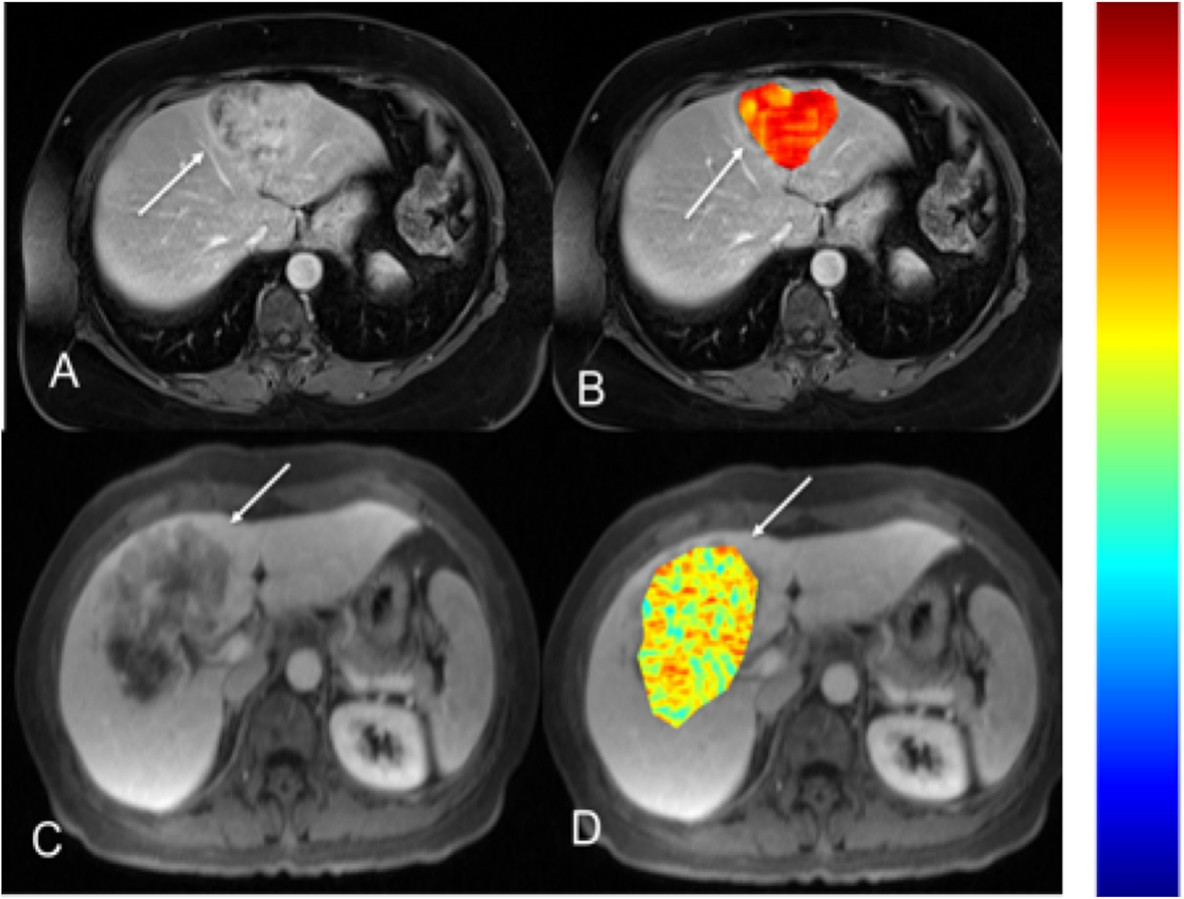
MRI texture feature of difference entropy found to be a significant predictor of TTR on univariable analysis. Top row (**a**, **b**): 54-year-old female with moderately differentiated ICC in segment 4 (AJCC stage 1). T1-weighted post-contrast MR image obtained during portal venous phase (**a**, arrow) with an overlying difference entropy texture map (**b**, arrow). This patient’s ICC difference entropy parameter was calculated to be 2.16 (scale 0–3, blue-red), with corresponding TTR of 825 days. Bottom row (**c**, **d**): 56-year-old female with poorly differentiated ICC (AJCC stage 3) in segment 4/8 on T1-weighted post-contrast MR image obtained during portal venous phase (**c**, arrow) and with an overlying difference entropy texture map (**d**, arrow). This patient’s ICC difference entropy parameter was calculated to be 1.22 (scale 0–3, blue-red) with corresponding TTR of 456 days. Color scale bar is shown on the right

Regarding outcomes, univariable analysis found that for both observers, the presence of satellite lesions on CT (HR 2.35 [1.16–4.76], *p* = 0.015; HR 3.27 [1.57–6.79], *p* = 0.001 for observers 1 and 2, respectively) and vascular involvement on MRI (HR 3.79 [1.46–9.84], *p* = 0.003; HR 4.17 [1.48–11.80], *p* = 0.004) were predictive of TTR. Biliary obstruction on CT (observer 2), vascular involvement on CT (observer 1), and satellite lesions on MRI (observer 1) were also predictive of TTR (all *p*-values < 0.035) in one observer. Enhancement pattern on MRI (HR 4.69 [1.12–19.67], *p* = 0.021) for observer 2 and satellite lesions on MRI (HR 3.32 [0.99–11.13], *p* = 0.039) for observer 1 were predictive of OS (Fig. 3). Results summarizing associations of qualitative imaging traits with outcomes on univariable analysis are shown in Table 5.

**Table 5:** Association of qualitative imaging features for CT and MRI provided by two independent observers with time to recurrence (TTR) and overall survival (OS) in patients with ICC. The hazard ratios (HR) and lower and upper limits of a 95% confidence interval from Cox regression are shown

	Feature	TTR							
		Observer 1				Observer 2			
		HR	95% CI	p*	p**	HR	95% CI	p*	p**
**CT**	Biliary obstruction	1.2	0.62–2.33	0.593	0.947	**2.22**	**1.14–4.36**	**0.017**	0.075
	Capsular retraction	1.11	0.57–2.18	0.758	0.631	1.03	0.50–2.10	0.938	0.791
	Enhancement pattern	1.44	0.50–4.11	0.498	0.458	1.72	0.66–4.49	0.264	0.202
	Satellite lesions	**2.35**	**1.16–4.76**	**0.015**	**0.008**	**3.27**	**1.57–6.79**	**0.001**	**< 0.001**
	Vascular involvement	**2.05**	**1.04–4.06**	**0.035**	**0.085**	1.77	0.92–3.42	0.083	0.122
**MRI**	DWI Appearance	1.26	0.31–5.09	0.742	0.603	2.05	0.54–7.73	0.281	0.148
	Biliary obstruction	1.64	0.65–4.15	0.288	0.338	1.88	0.75–4.75	0.174	0.122
	Capsular retraction	0.75	0.30–1.88	0.537	0.498	0.61	0.18–2.13	0.437	0.321
	Enhancement pattern	0.55	0.13–2.38	0.414	0.677	2.04	0.66–6.27	0.206	0.196
	Signal on HBP	1.01	0.83–1.23	0.925	0.888	1.04	0.85–1.26	0.727	0.718
	Satellite lesions	**8.3**	**2.60–26.52**	**< 0.001**	**< 0.001**	2.24	0.64–7.91	0.198	0.163
	Vascular involvement	**3.79**	**1.46–9.84**	**0.003**	**0.005**	**4.17**	**1.48–11.80**	**0.004**	**0.004**
	**Feature**	**OS**							
		**Observer 1**				**Observer 2**			
		**HR**	**95% CI**	p*	p**	**HR**	**95% CI**	p*	p**
**CT**	Biliary obstruction	0.74	0.35–1.57	0.427	0.471	0.99	0.46–2.13	0.981	0.9
	Capsular retraction	0.7	0.33–1.52	0.366	0.327	0.81	0.34–1.92	0.626	0.47
	Enhancement pattern	1.41	0.32–6.29	0.654	0.783	1.38	0.31–6.20	0.673	0.656
	Satellite lesions	1.86	0.85–4.07	0.113	0.315	2.13	0.96–4.70	0.056	0.117
	Vascular involvement	1.18	0.54–2.55	0.68	0.886	0.73	0.33–1.61	0.436	0.702
**MRI**	DWI Appearance	1.41	0.23–8.60	0.709	0.335	1.93	0.32–11.62	0.464	0.166
	Biliary obstruction	1.04	0.33–3.27	0.953	0.72	1.98	0.63–6.26	0.234	0.617
	Capsular retraction	1.09	0.35–3.39	0.88	0.622	0.89	0.19–4.07	0.879	0.784
	Enhancement pattern	1.86	0.50–6.90	0.346	0.186	**4.69**	**1.12–19.67**	**0.021**	**0.028**
	Signal on HBP	1.33	0.88–2.00	0.142	0.085	1.36	0.90–2.04	0.111	0.064
	Satellite lesions	**3.32**	**0.99–11.13**	**0.039**	0.255	3.47	0.89–13.53	0.056	0.327
	Vascular involvement	1.93	0.62–6.02	0.252	0.66	2.82	0.89–8.89	0.065	0.126

Footnote: *p-values from the log-rank test, **p-values from generalized Wilcoxon tests

Significant results are bolded

### Quantitative image analysis

There was no quantitative measurement that was predictive of poor tumor differentiation (all *p*-values > 0.13). Multiple texture features were associated with higher AJCC stage, including CT energy (AUC 0.78, *p* = 0.009), CT entropy (AUC 0.80, *p* = 0.002), CT information measure of correlation 1 (AUC 0.79, *p* = 0.002), CT information measure of correlation 2 (AUC 0.79, *p* = 0.002), MRI contrast (AUC 0.74, *p* = 0.030), MRI correlation (AUC 0.73, *p* = 0.041), MRI homogeneity (AUC 0.74, *p* = 0.023), MRI difference variance (AUC 0.74, *p* = 0.030), and MRI difference entropy (AUC 0.73, *p* = 0.030). Enhancement ratios on both CT and MRI were not associated with either tumor grade or AJCC stage (all *p*-values > 0.124).

The mean lesion size on imaging was 6.8 ± 3.4 cm (range 1.4–16.0 cm). Larger tumor size was a significant predictor for TTR (HR 1.12, *p* = 0.001) and OS (HR 1.10, *p* = 0.028) on univariable analysis, and remained a significant predictor of TTR on multivariable analysis (HR 1.13 [1.04–1.22], *p* = 0.003).

**Fig. 4:**
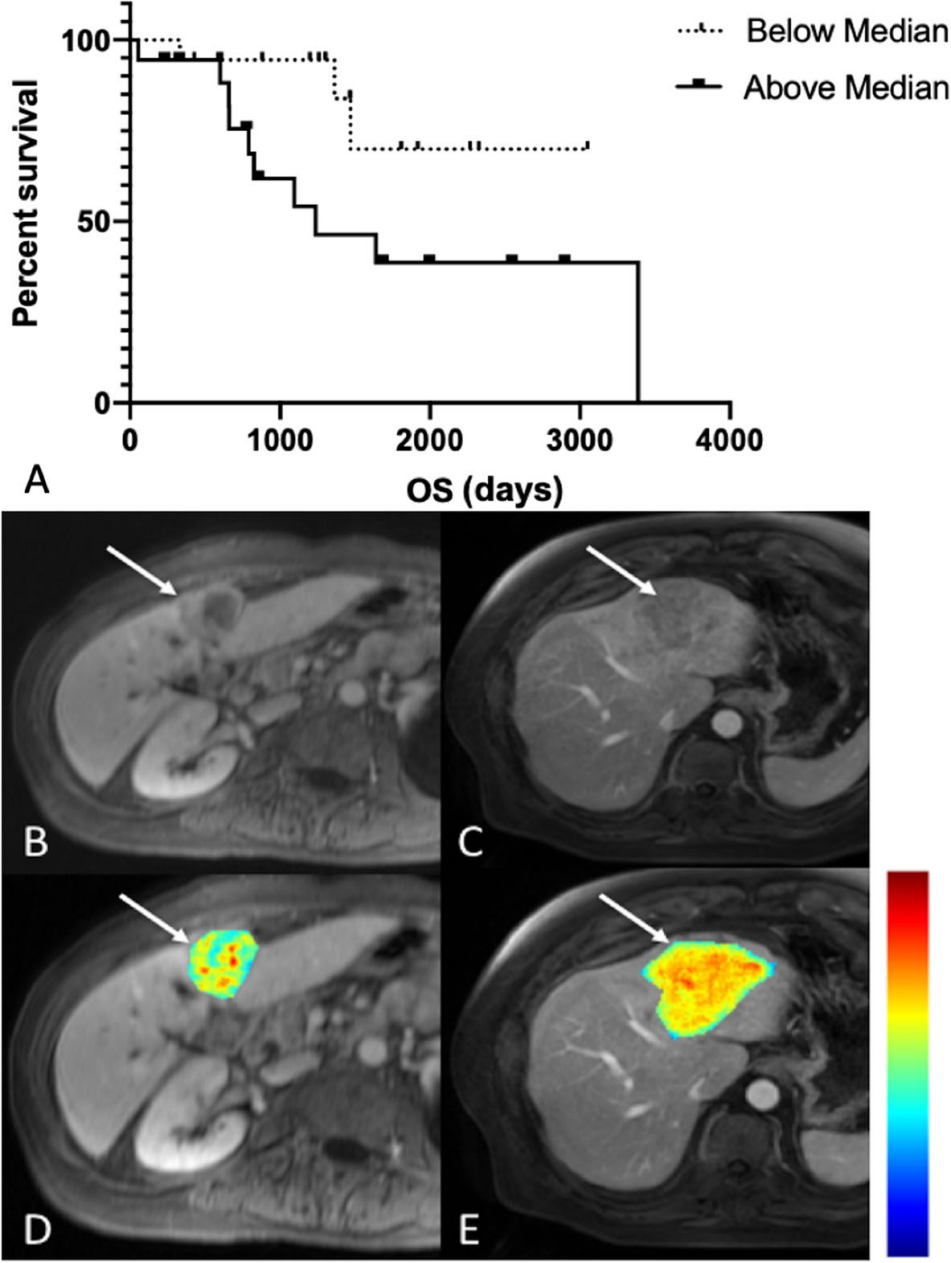
MRI texture feature information measure of correlation 1 found to be a significant predictor of OS on univariable analysis. Kaplan-Meier curve for OS as a function of whether MRI information measure of correlation 1 was above (solid line) or below (dashed line) its median value of − 0.458 (**a**). 68-year old female with poorly differentiated ICC in segment 4 (AJCC stage 3) on T1-weighted post-contrast MR image obtained during portal venous phase (**b**, arrow) with an overlying information measure of correlation 1 texture map (**d**, arrow). This patient’s information measure of correlation 1 was calculated to be − 0.554 (scale − 1.0-0.0, blue-red), with corresponding OS of 1259 days. 63-year old male with moderately differentiated ICC in segment 4A/2 (AJCC stage 3) on T1-weighted post-contrast MR image obtained during portal venous phase (**c**, arrow) with an overlying information measure of correlation 1 texture map (**c**, arrow). This patient’s information measure of correlation 1 was calculated to be − 0.330 (scale − 1.0-0.0, blue-red), with corresponding OS of 658 days. Color scale bar is shown on the right

In the univariable analysis, ADCmean (*p* = 0.042) was found to be an independent predictor of TTR on the log-rank test (*p* = 0.042), but was not found to be significant on subsequent Cox proportional hazards regression (*p* = 0.571). ADCmin was found to be an independent predictor of OS on the log-rank (*p* = 0.005) and generalized Wilcoxon tests (*p* = 0.015), but was not found to be statistically significant on subsequent Cox proportional hazards regression (*p* = 1.0), likely related to sample size (*n* = 19). For enhancement ratios (ERs), only arterial phase (AP) ER on MRI was associated with TTR (HR 0.34 [0.12–1.0], *p* = 0.024). ERs on both CT (AP and PVP) and MRI (AP, PVP, HBP) were not predictive of OS (all *p*-values > 0.104). Several texture features were associated with TTR, including CT sum average (HR 0.70 [0.49–0.99], *p* = 0.021), CT entropy (HR 1.59 [1.03–2.45], *p* = 0.038), CT information measure of correlation 2 (HR 0.73 [0.53–1.00], *p* = 0.047), MRI difference entropy (HR 0.58 [0.37–0.91], *p* = 0.013) and MRI homogeneity (HR 1.79 [1.13–2.84], *p* = 0.006) (Fig. 3). The only texture feature associated with OS was MRI information measure of correlation 1 (HR 1.87 [0.97–3.62], *p* = 0.038) (Fig. 4). Results summarizing associations of quantitative imaging features with outcomes on univariable analysis are shown in Table 6.

**Table 6:** Association of quantitative imaging features for CT and MRI with time to recurrence (TTR) and overall survival (OS) in patients with ICC. The hazard ratios (HR) and lower and upper limits of a 95% confidence interval from Cox regression are shown, when available

	Feature	TTR				OS			
		p*	p**	HR	95% CI	p*	p**	HR	95% CI
**CT**	Energy	0.096	0.065	0.51	0.23–1.15	0.345	0.293	0.65	0.27–1.59
	Contrast	0.713	0.980	1.06	0.77–1.46	0.305	0.278	1.21	0.84–1.74
	Correlation	0.864	0.875	0.97	0.71–1.34	0.367	0.335	0.85	0.59–1.22
	Variance	0.208	0.185	0.83	0.63–1.11	0.184	0.243	0.80	0.58–1.11
	Homogeneity	0.404	0.496	0.87	0.62–1.22	0.385	0.378	0.83	0.54–1.27
	Sum Average	**0.045**	**0.021**	**0.70**	**0.49–0.99**	0.147	0.132	0.72	0.46–1.12
	Sum Variance	0.124	0.126	0.79	0.58–1.07	0.136	0.100	0.76	0.53–1.09
	Sum Entropy	0.153	0.089	1.43	0.88–2.33	0.551	0.567	1.19	0.68–2.06
	Entropy	**0.038**	**0.042**	**1.59**	**1.03–2.45**	0.292	0.247	1.32	0.79–2.21
	Difference Variance	0.713	0.980	1.06	0.77–1.46	0.305	0.278	1.21	0.84–1.74
	Difference Entropy	0.387	0.527	1.15	0.83–1.60	0.304	0.292	1.23	0.83–1.83
	Information Measure of Correlation 1	0.064	0.075	1.49	0.98–2.28	0.421	0.331	1.23	0.75–2.02
	Information Measure of Correlation 2	**0.047**	0.132	**0.73**	**0.53–1.00**	0.187	0.209	0.78	0.54–1.13
	Maximal Correlation Coefficient	0.165	0.169	0.77	0.53–1.11	0.213	0.174	0.74	0.47–1.18
	Enhancement Ratio (AP)	0.104	0.146	0.43	0.16–1.2	0.361	0.320	0.63	0.23–1.70
	Enhancement Ratio (PVP)	0.238	0.247	0.64	0.30–1.4	0.187	0.127	0.57	0.25–1.30
**MRI**	Energy	0.922	0.909	1.02	0.66–1.59	0.520	0.543	0.73	0.29–1.88
	Contrast	0.133	0.102	0.63	0.34–1.15	0.097	0.101	0.48	0.20–1.15
	Correlation	0.129	0.097	1.58	0.88–2.86	0.087	0.091	2.05	0.89–4.74
	Variance	0.601	0.641	0.89	0.58–1.37	0.654	0.750	0.87	0.48–1.59
	Homogeneity	**0.011**	**0.006**	**1.79**	**1.13–2.84**	0.171	0.191	1.46	0.84–2.54
	Sum Average	0.277	0.194	0.79	0.51–1.22	0.703	0.766	0.90	0.52–1.56
	Sum Variance	0.629	0.584	0.90	0.60–1.37	0.838	0.877	0.94	0.54–1.65
	Sum Entropy	0.240	0.192	1.43	0.78–2.62	0.354	0.465	1.48	0.65–3.39
	Entropy	0.323	0.322	1.28	0.79–2.08	0.186	0.199	1.60	0.80–3.22
	Difference Variance	0.133	0.102	0.63	0.35–1.15	0.097	0.101	0.48	0.20–1.15
	Difference Entropy	**0.015**	**0.013**	**0.58**	**0.37–0.91**	0.140	0.154	0.67	0.38–1.16
	Information Measure of Correlation 1	0.420	0.524	1.21	0.76–1.92	0.059	**0.038**	**1.87**	**0.97–3.62**
	Information Measure of Correlation 2	0.736	0.985	0.93	0.61–1.43	0.068	0.050	0.62	0.36–1.05
	Maximal Correlation Coefficient	0.464	0.642	1.15	0.79–1.65	0.621	0.586	1.15	0.67–1.97
	ADCMean	**0.042**	0.058	NA	NA	0.309	0.289	NA	NA
	ADCMin	0.476	0.504	NA	NA	**0.005**	**0.015**	NA	NA
	Enhancement Ratio (AP)	**0.040**	**0.024**	**0.34**	**0.12–1.00**	0.476	0.569	0.64	0.19–2.2
	Enhancement Ratio (EP)	0.439	0.232	0.69	0.26–1.80	0.536	0.688	0.70	0.22–2.2
	Enhancement Ratio (HBP)	0.117	0.119	NA	NA	0.522	0.495	NA	NA
	Enhancement Ratio (PVP)	0.069	0.058	0.42	0.17–1.10	0.226	0.318	0.52	0.18–1.50

Footnote: *p-values from the log-rank test, **p-values from generalized Wilcoxon tests

Significant results are bolded

Results of the multivariable analysis demonstrated that after adjusting for CA 19–9 and tumor size, satellite lesions on CT (HR 2.79 [1.09–7.15], *p* = 0.032) for observer 2, vascular involvement on MRI for observer 1 (HR 0.10 [0.01–0.85], *p* = 0.035), and MRI variance (HR 0.55 [0.31–0.97], *p* = 0.040) were predictive of TTR. No qualitative or quantitative feature was predictive for OS when adjusted for CA 19–9 and lymph nodes (all *p*-values > 0.088). There was no set of two or more imaging measures that were significant independent predictors of tumor grade, AJCC stage, TTR, or OS after adjusting for the competing risk factors identified for that outcome.

## Discussion

In this study, we tested qualitative and quantitative imaging data obtained from pre-operative CT and/or MRI as well as CA 19–9 with pathology and outcomes in 73 patients with ICC. Our median TTR of 53.9 months (IQR 73.2 months, range 1.6–99 months) and median OS of 79.7 months (IQR 75.4 months, range 1.8–137.3 months) are longer than most published reports (median TTR and median OS have previously been reported as ranging from 7 to 34 months and 21.8–49 months, respectively) [1, 10, 33, 34]. Nevertheless, our findings of elevated serum CA 19–9, histopathologic vascular invasion, metastatic lymph nodes, AJCC tumor stage, and tumor size (measured at imaging) as significant predictors of TTR and OS agree with the literature [1, 2, 13].

We demonstrated fair accuracy for prediction of higher AJCC stage (maximal AUC of 0.73 for biliary obstruction on CT, *p* = 0.002, observer 2 and AUC 0.73 of 0.73 for vascular involvement on MRI, *p* = 0.01, observer 2) and tumor grade (maximal AUC of 0.68 for biliary obstruction on MRI, *p* = 0.032, observer 1) utilizing qualitative and quantitative image analysis. After adjusting for competing risk factors using multivariable analysis, we found that the presence of satellite lesions on CT (HR 2.79 [1.09–7.15], *p* = 0.032, observer 2), vascular involvement on MRI (HR 0.10 [0.01–0.85], *p* = 0.035, observer 1), and the texture feature MRI variance (HR 0.55 [0.31–0.97], *p* = 0.040) remained predictors of TTR. Several quantitative imaging features, including some Haralick texture features in addition to other qualitative imaging traits, were significant predictors of TTR and OS in univariable analysis, but were not confirmed at multivariable analysis. We believe these results are promising, especially as data regarding texture analysis for noninvasive characterization of ICC and clinical outcomes using cross-sectional imaging is limited [22].

Our findings corroborate and expand upon previous studies that have investigated the imaging features of ICC and the correlations between radiologic and pathologic findings [2, 13, 16]. The imaging features of ICC correlate with specific histopathologic features: intrahepatic biliary dilatation reflects the tumor’s origin from the biliary duct; peripheral enhancement represents viable tumor cells, with delayed enhancement of a central fibrous/scirrhous stroma composed of desmoplastic tissue occurring later in time; and the presence of satellite nodules indicates the tumor’s proclivity to invade small portal vessels and along portal triads [8]. Previous work has identified a prognostic implication of the delayed phase enhancement on CT, with greater degree of enhancement representing a greater quantity of fibrous stroma and perineural invasion, correlating with poorer outcomes [14]. The presence of satellite nodules, macrovascular invasion, and portal venous or delayed phase enhancement has been previously described as poor prognostic indicators [2, 8, 11, 13, 14, 35, 36]. Of note, some qualitative features were statistically significant for only one of the two observers, likely due to our limited sample size; as a result, features there were not significant for both observers may therefore not be helpful in predicting outcomes. ADCmean has been shown to be significantly lower than that of the adjacent liver parenchyma in ICC (with poorly differentiated tumors demonstrating a significantly lower ADCmean); our findings of ADCmean as an independent predictor of TTR on the log-rank test (*p* = 0.042), and ADCmin as an independent predictor of OS on the univariable log-rank (*p* = 0.005) and generalized Wilcoxon (*p* = 0.015) tests support the potential value of DWI in the imaging workup of ICC, while notably, the lack of significant results using other statistical tests could be explained by small sample size [17, 18].

Radiomics quantification including histogram quantification and Haralick texture analysis, a mathematical method that generates various quantitative parameters characterizing the spatial variation of gray levels throughout an image, has shown correlations between calculated texture features and histopathologic characteristics, genomic data, and clinical outcomes in various tumor types [24, 27, 28, 37–39]. Texture analysis is sensitive to subtle changes in tumor morphology that may not be detected visually. Intra-tumoral changes due to neovascularity, tumor necrosis, and aggressive growth patterns within ICC contribute to heterogeneity, which may be quantified using texture analysis. As expected in our study, the texture features found to be significant on MRI were different than those found to be significant on CT without redundancy or overlap, reflecting the innate differences between both modalities. The MRI texture feature variance was the only texture feature to remain a significant predictor of outcomes on multivariable analysis; this may suggest that MRI texture features are potentially more valuable than CT texture features in characterizing ICC, possibly due to the greater soft tissue contrast resolution in MRI.

These specific texture features in our study have also shown significant results for other tumor types. For example, the entropy feature, which is a measure of disorder in the distribution of signal intensities in the ROI and is thought to be a manifestation of tumor heterogeneity, has been previously shown to predict tumor recurrence, disease free survival and OS for hepatocellular carcinoma (HCC) [40, 41]. In a recent study of 25 patients with ICC with biopsy, significant correlations between certain grey level co-occurrence matrix textures features based on CT and immunohistochemical markers of hypoxia were identified [22]. Specifically, the entropy texture feature was significantly associated with EGFR expression (R^2^ = 0.17, *p* < 0.05). The authors also found that the correlation texture feature was associated with VEGF expression (R^2^ = 0.23, *p* < 0.05) and EFGR expression (R^2^ = 0.21, *p* < 0.05); in contrast, we did not find any associations between the correlation texture feature on either CT or MRI and pathologic markers or outcome [22]. While previous studies including ours have described associations between imaging texture features and pathologic features and clinical outcomes, establishment of meaningful biologic correlates for specific texture features remains under active investigation.

Our methods could be clinically applicable and relevant, especially as this type of analysis can be performed using a standard clinical CT or MRI protocol for the initial preoperative assessment of liver tumors in efforts to predict tumor type, tumor grade, tumor stage, and outcomes. Imaging features can be useful to predict TTR so that more aggressive neoadjuvant and/or locoregional therapies—including chemotherapy—and postoperative surveillance can be instituted. Furthermore, integration of clinical variables, especially serum CA 19–9, in conjunction with qualitative and quantitative imaging data may potentially yield the best predictive accuracy for the non-invasive assessment of ICCs. Regarding texture analysis, there does need to be standardization of sequences, protocols, and radiomics analysis to enable widespread application of this technique.

We recognize several limitations to our study. Several features in our qualitative analysis were combined in order to provide enough statistical power; expansion of our sample size in subsequent work would allow for a more robust statistical analysis. There was variability in the CT and MRI acquisition techniques as these exams were performed on a variety of clinical scanners over the duration of the long study period. A window of obtaining preoperative imaging up to 6 months prior to surgery may have introduced bias and affected our results as the tumor could have developed more aggressive features by the time surgery was performed. Only one observer was included for the quantitative imaging analysis; having two independent observers would have allowed for assessment of inter-observer reproducibility of quantitative assessments. Assessment of DWI/ADC was limited as there were only 19 cases where DWI/ADC was available, and variation in image acquisition technique can influence ADC measurement. We sought to minimize this bias by analyzing DWI acquired with the same b-values (b50 and b400–500 s/mm^2^), however more focused work on DWI/ADC with less potential for bias is needed to assess its true ability in predicting outcomes. Despite differences in texture-based discrimination existing between 1.5 T and 3 T MRI due to varying SNR and artifacts at different field strengths, our relatively low number of 3 T (*n* = 4) as compared with 1.5 T (*n* = 32) scans, as well as our efforts to normalize the texture data, minimizes this limitation. It is possible that our limited sample size and variability due to scanners and protocols used in this retrospective study may explain why combinations of blood biomarkers and imaging features did not yield significant results at multivariable analysis. Finally, our study lacks a validation cohort, which may be assessed in future studies.

## Conclusion

In conclusion, our study demonstrated reasonable accuracy for the prediction of tumor grade and higher AJCC stage in ICC utilizing certain qualitative and quantitative imaging traits. Serum CA 19–9, imaging tumor size, presence of metastatic lymph nodes, and qualitative imaging traits of satellite lesions and vascular involvement are predictors of patient outcomes, along with a promising predictive ability of certain quantitative texture features. Identification of imaging traits that are markers of outcome in patients with ICC may be valuable for treatment planning. Future directions include verification of these findings, correlation with molecular profiling of ICC tumors, and possibly integration of FDG PET/MRI for assessment of ICC aggressiveness and outcome.

## Data Availability

All data generated or analyzed during this study are included in this published article [and its supplementary information files].

## Abbreviations

ADC: Apparent diffusion coefficient
DWI: Diffusion weighted imaging
ER: Enhancement ratio
HBP: Hepatobiliary phase
HR: Hazard ratio
ICC: Intrahepatic cholangiocarcinoma
OS: Overall survival
ROI: Region of interest
SI: Signal intensity
TTR: Time to recurrence
